# Cultural values shape the expression of self-evaluative social emotions

**DOI:** 10.1038/s41598-021-92652-8

**Published:** 2021-06-23

**Authors:** Antje von Suchodoletz, Robert Hepach

**Affiliations:** 1 Department of Psychology, New York University Abu Dhabi, PO Box 129188, Abu Dhabi, UAE; 2 University of Oxford, Oxford, UK

**Keywords:** Human behaviour, Psychology

## Abstract

Social emotions are key to everyday social life and therefore shaped by cultural values in their expression. Prior research has focused on facial expressions of emotions. What is less clear, however, is the extent to which cultural values shape other modalities of emotional expression. In the present study, we applied a novel paradigm using depth sensor imaging technology to capture changes in participants’ body posture in real time. We aimed to (1) identify the nuances in the postural expression that are thought to characterize social emotions and (2) assess how individual differences in cultural values impact the postural expression of emotions. Participants in two separate studies were 132 undergraduate college students whose upper-body postural expansion was recorded after they recalled emotion episodes. Positive emotions elevated participants’ upper-body posture whereas negative emotions resulted in lowered upper-body posture. The effects on changes in upper-body posture were moderated by participants’ self-ratings of the vertical and horizontal dimensions of individualism and collectivism. The findings provide initial evidence of the nuances in the way cultural values influence the postural expression of emotions.

The capacity for culture is a hallmark characteristic of human sociality^1^. The psychological shape and breadth of human culture is based human-unique cooperative cognitions stemming from joint commitments to shared intentionality^2^. Virtually all major theories on human development, including Lev Vygotsky’s Sociocultural Theory, Barbara Rogoff’s Transformation of Participation Perspective, and Urie Bronfenbrenner’s Bioecological Theory of Human Development, argue that the cultural context crucially shapes everyday experiences and social interactions with others^3^. When examining cultural mechanisms of human functioning, psychologists typically assessed aspects such as shared rules of social behavior and social institutions (i.e., “social culture”) and shared values and beliefs in a human group (i.e., “subjective culture”) (p. 646)^4^.

Cross-cultural psychological research has shed light on cultural differences in behavior, cognitions, and emotions of people from different cultural backgrounds, predominately comparing Westerners and (East) Asians^4–8^. Differences have been found in perceptual processes, e.g., Westerners analyze an object of interest independent of its context whereas Asians focus on the relationship between the object and its context^7^. Such findings have been attributed to differences in cultural values regarding views of the self. Individuals from Western cultural backgrounds emphasize one’s independence, uniqueness, and autonomy; “The Western self is composed of fixed attributes and can move from one setting or context to another without significant alteration.” (p. 11,164)^6,9^. In contrast, individuals from Asian cultural backgrounds focus on a person’s interdependence, thus situating oneself in relation to significant others resulting in a view of the self that is highly dependent on the social context^6,9^. Individuals’ behavior, cognitions, and emotions are guided by such cultural values whereby social-contextual opportunities or constraints influence the strength and direction of the association^10^. With regard to emotions, social-contextual factors influence the desirability of emotions, the way in which emotions are expressed, and the (perceived) appropriateness of emotional expressions^11–13^.

An established (predominately Western) research tradition viewed people solely in terms of their membership in cultural groups and contrasted these groups on the basis of differences in the relative emphasis placed on independence (labeled individualistic) versus interdependence (labeled collectivistic)^14,15^. Yet, cultural values, such as independence and interdependence, may coexist within individuals^15^. It is, therefore, critical to assess variation in cultural values at the individual level to isolate “the ‘active ingredients’ in cross-cultural differences” (p. 41)^15^. Methodologically, such an approach requires the assessment of elements of individualism and collectivism separately, ideally using multiple scales that tap a single specific dimension of individualism and collectivism^15^. To capture variation at the individual level, Singelis and colleagues^16^ developed a measure to assess different dimensions, i.e., vertical and horizontal, of individualism and collectivism that are based on Triandis’ work^17^. The horizontal dimension is focused on equality, i.e., the status of one’s own self is equal to those of others. In contrast, the vertical dimension refers to inequality between one’s own self and the self of others, i.e., people do not see each other as the same^16^. Thus, horizontal individualism is focused on one’s independence (i.e., the desire to be unique and distinct). One’s own status (i.e., worth, dignity, and rights) is seen as equal to those of others. Vertical individualism is also focused on one’s uniqueness but the reference to hierarchical or subordinate social relations leads to competition with others in order to become distinguished and acquire status. Individuals who emphasize horizontal and collectivistic practices view the status of their self as similar to that of others and prioritize common goals, interdependence, and sociability. Vertical collectivism is focused on loyalty to one’s own group and interdependence, but in contrast to horizontal collectivism, individuals who emphasize vertical and collectivistic practices adhere to hierarchical or subordinate social relations within their group^18^. The vertical-horizontal, individualism-collectivism typology can crucially disentangle sources of cultural variation^13^. In contrast to previous findings on how culture influences verbal self-report measures, much less is known about whether and how culture impacts *non-verbal* measures of cultural identity such as experienced (and expressed) social emotions.

Emotions, in particular social emotions, are powerful communicative commitment devices that regulate social interactions^19–21^. The classification of emotions as social is based on the nature of its underlying core evaluation or appraisal. Because social emotions necessarily depend on other people, either directly (experiencing or imagining their thoughts, feeling or actions) or indirectly (considering social norms or conventions), appraisals implicitly or explicitly refer to social factors and an event’s relevance to such factors^22^. The experience and expression of social emotions is thus dependent upon one’s appraisal of social factors whereas this is less the case for other emotions^22^. According to componential emotion models, an “appraisal is […] considered the central mechanism in the elicitation and differentiation of emotion. […] Emotions characterized by different appraisal profiles are expected to have different expression patterns.” (p. 1087)^23^.

Appraisal processes relate to the informational functions of emotions. Emotions provide information that is vital for successfully navigating one’s social life, that is, information about feelings, desires, motivations, and intentions^12,13^. Social-functional approaches to emotion argue that the information is both, intrapersonal (i.e., relevant to the self) and interpersonal (i.e., relevant to observers)^12^. The informational content is shaped by social-contextual factors, such as cultural norms and values^12,13^. For example, Matsumoto and colleagues^24,25^ argue that individuals from Western cultural contexts place a greater emphasis on the intrapersonal meaning of emotions. That is, emotions are viewed as important personal experiences that promote uniqueness, separateness, and autonomy, all of which are important cultural values of independence. In contrast, individuals from Asian cultural contexts emphasize the interpersonal meaning of emotions. Emotions are seen as interactive experiences that promote cultural values of interdependence, i.e., harmony and cooperation with significant others. The different foci correspond with prevailing conceptualizations of emotions^26^. Consistent with the assumptions underlying the distinction between independence versus interdependence, prior research suggests that individuals from Western cultural contexts understand emotions as primarily “residing within people” whereas individuals from Asian cultural contexts view emotions “as residing between people” (p. 1428)^26^. For example, it was found that the reported frequency of positive emotions was related to the frequency of interpersonally disengaged positive emotions for Westerners but with interpersonally engaged emotions for Asians^5^. This is consistent with findings that individuals from Asian cultural contexts are more likely to experience and express emotions in relational contexts. In contrast, individuals from Western cultural contexts are more likely to experience and express emotions when the focus is on the individual^26^.

Taken together, emotions crucially regulate human social interactions^27,28^ and individuals rely on various modalities (facial expression, prosody, body motion, posture) when expressing emotions^29,30^. Building on Ekman’s seminal work^31,32^, prior research has focused on facial expressions of emotions. More recently, the literature has called for greater attention to other modalities of emotional expression^33^. In particular, research on the affective body context has emerged rapidly, recognizing body posture as an important medium for emotional expression^23,29,30^. In fact, when adults are asked to identify emotions from combinations of facial expression and body posture, it is body posture that trumps facial expression^29,34–37^.

Postural changes are reliably identifiable from a person’s gait^38^ as well as whole-body movement^23^. Most previous empirical work had adult raters code posture changes from video recordings^38–40^, photographs^41–43^, drawings^44^, and computer-animated mannequins or point light displays^45,46^. What is crucially needed, however, is a reliable automated and objective measure of body posture that allows us to (1) identify the nuances in the expression that are thought to characterize social emotions and (2) assess how individual differences in cultural values impact the expression of emotions, specifically postural expression. In the present study, we applied a novel paradigm using depth sensor imaging technology to capture changes in participants’ body posture in real time^47–49^. Specifically, we recorded adult participants’ upper-body posture in response to recalling distinct emotional episodes and we explored effects of participants’ self-reported cultural values on their respective postural expression. Informed by emotion theory, we focused on social emotions because they are associated with mentally representing another person’s thoughts, feelings and/or action to an experience^22,50^ and their expression, therefore, is thought to be regulated by how cultural values shape one’s view of the self in relation to others. We selected two common and prototypical social emotions: one of positive valence, pride, and one of negative valence, shame (for review^22^). We further included two other emotions that matched the social emotions in valence but are not considered to be *social*: one with positive valence, joy, and one with negative valence, disappointment. The inclusion of these more basic emotions allowed us to test if the variation in the postural expression is specific to social emotions—and not any positive or negative emotion.

Based on previous findings on self-reported cultural values, our specific research aims were as follows: (1) to explore the unique expression of social emotions via changes in participants’ live posture and (2) to test how cultural values along the vertical-horizontal, individualism-collectivism typology relate to changes in the postural expression of social emotions. First it is important to analyze if postural expression allows for emotion differentiation, i.e., conveys emotion-specific information. Past studies found that slouch postures relate to negative emotions whereas erect postures relate to positive emotions^51^. We therefore investigated differences in the postural expression between the four selected emotions, i.e., pride, shame, joy and disappointment, independent of whether they are social or not, and expected positive emotions (pride and joy) to result in an increase in upper-body posture and negative emotions (shame and disappointment) to result in a decrease in postural elevation. Furthermore, we tested whether the absolute difference in postural expression between the positive (pride) and negative (shame) social emotion can be explained by the general expected difference between positive and negative emotions. Second, we explored whether change in postural expression of social emotions is influenced by participants’ cultural values. Because of the lack of prior research, we did not formulate specific hypotheses for each of the individualism-collectivism dimensions. Furthermore, we explored whether effects of cultural values on the postural expression of social emotions was explained by effects of the same cultural values on the postural expression of other emotions to help the interpretation of the results with regard to cultural variation in the postural expression of social emotions.

To investigate body posture differences, we recorded participants’ upper-body postural expansion in response to imagery of two social emotions of negative and positive valence and two other emotions matching the valence of the social emotions. These analyses were preregistered. We ran two additional analyses on participants’ lower body posture to determine whether effects were specific to changes in upper-body posture. Participants were 132 undergraduate college students studying abroad. Students were recruited from an international university that enrolls students from more than 100 countries. We collected two different samples from the same population of students, one in April/May 2018 (Study 1) and one in November/December 2018 (Study 2). Changes in participants’ body posture were measured using a Microsoft Kinect depth sensor camera (see Fig. 1). The Kinect was controlled using series of Matlab-routines which track 20 skeletal points per frame for up to two individuals present in the frame. The code is publicly available (Github-link masked for review). To capture participants’ posture, we asked them to walk toward the Kinect camera at the beginning of the study (baseline phase) and following a series of emotion inductions (test phase). We calculated the change in postural elevation from baseline to each of the emotion elicitation trials. We ran 2 identical separate studies (*n* = 66 each) to replicate the findings on participants’ posture. In addition, we combined data from both studies to investigate individual differences in cultural variables on emotional (postural) expressiveness. Both studies were preregistered on the open science framework: Study 1: https://osf.io/9qbpw; Study 2: https://osf.io/4cjga.

**Figure 1:**
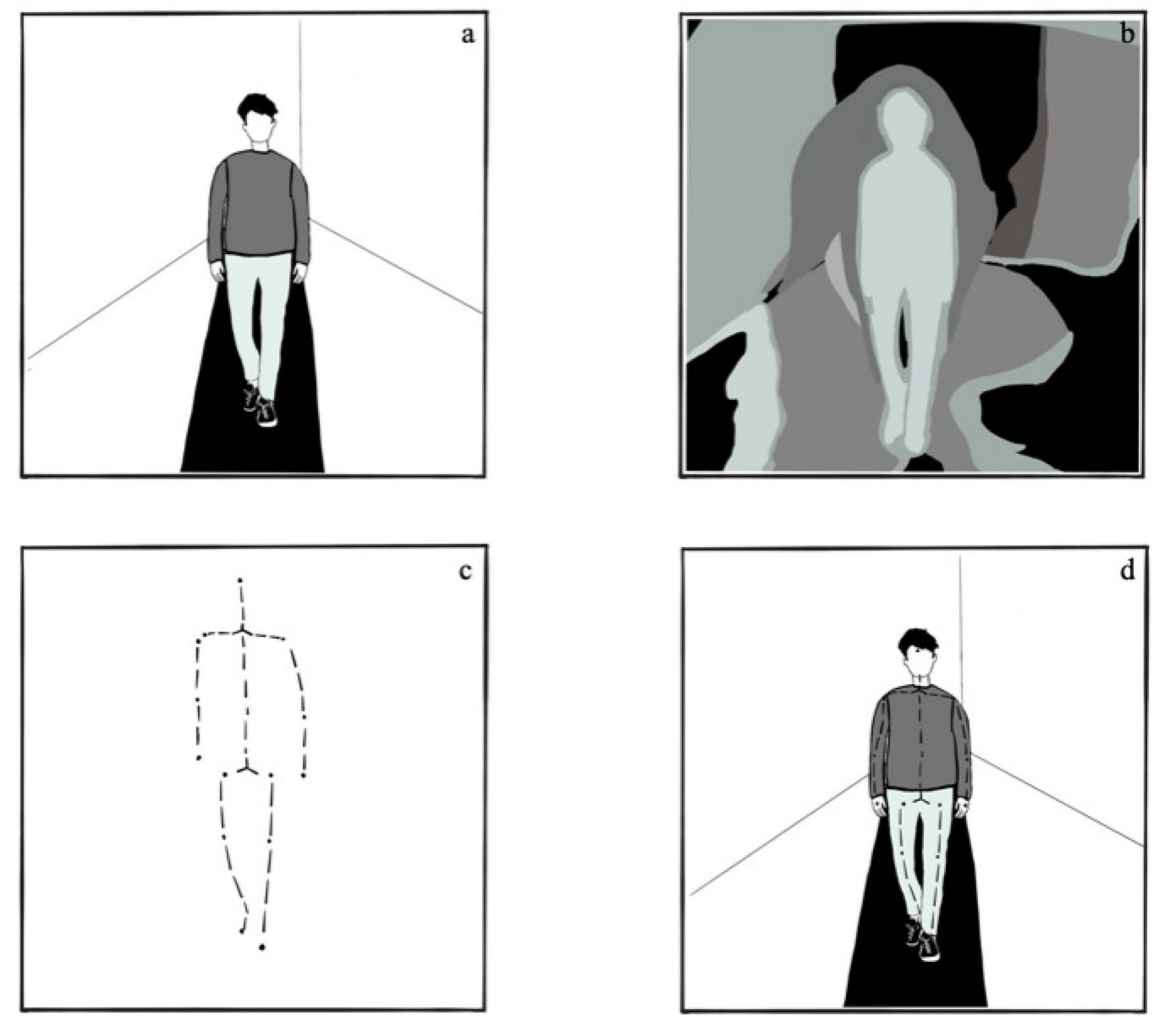
An illustration of the data captured by the Kinect motion sensor imaging technique. (**a**) RGB image (similar to regular video camera), (**b**) depth contour image, (**c**) estimated skeletal joints, (**d**) mapping of the estimated skeletal joints onto the RGB image. This study only used the output as shown in image (**c**). Illustration from Leonore Blume (https://noblu.de/).

Using a within-subjects design, participants completed a total of 10 trials: 2 baseline trials and 2 test trials for each of the following emotions: pride, shame, joy, and disappointment. The baseline trials were always presented first while the trials for each emotion were blocked in trials of 2. The presentation of the 4 emotion blocks was randomized (using randomize.org). In the baseline trials, participants were asked to walk toward the Kinect in a relaxed natural manner (see Fig. 2).

**Figure 2:**
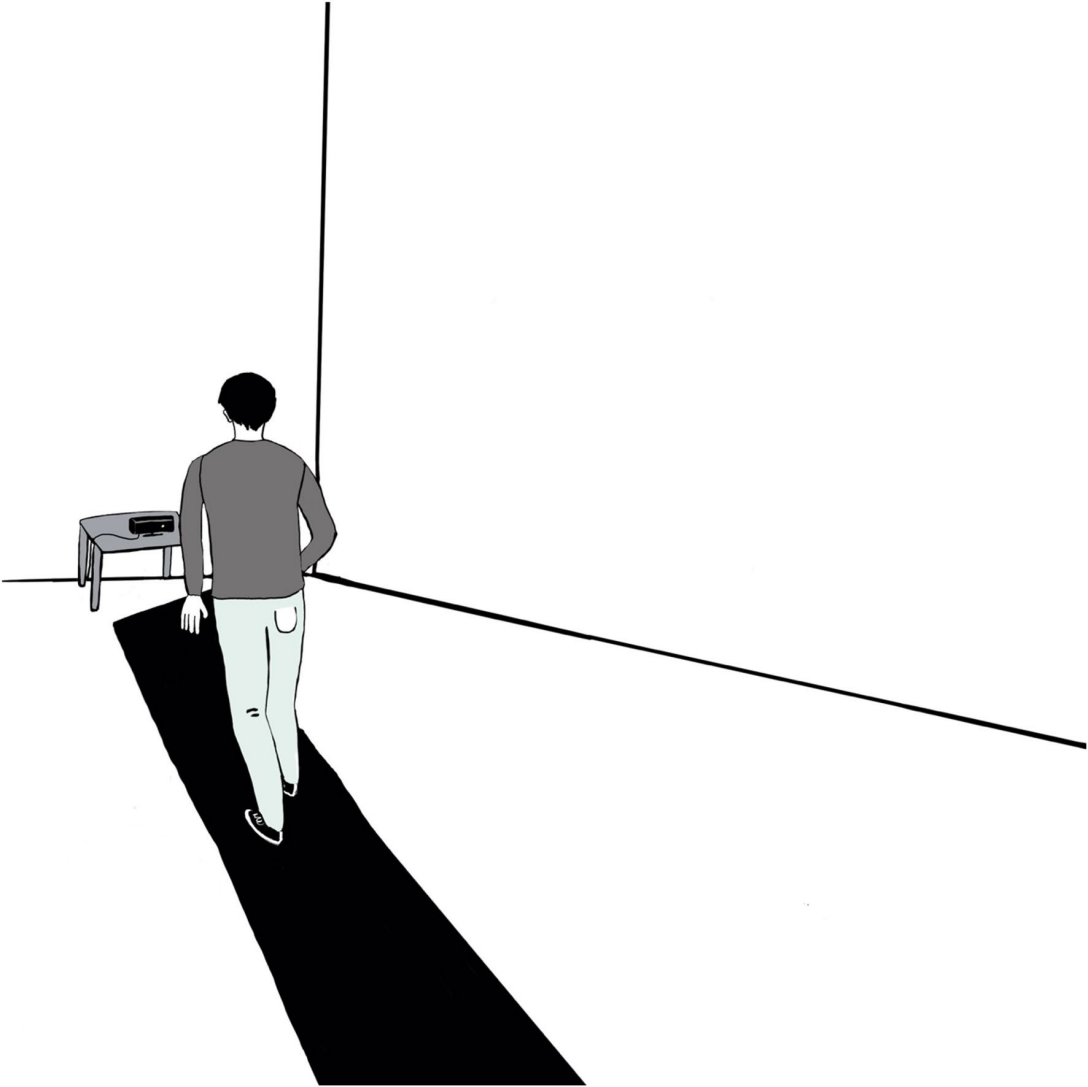
Illustration of the scene when participants walked toward the Kinect camera. Illustration from Leonore Blume (https://noblu.de/).

In the test trials, they were asked to recall an emotional experience and then walk toward the Kinect. Per emotion we captured two successive walks. Following each set of emotion walks, participants wrote down the event they recalled on a paper. After their final, 10th, walk subjects were asked to complete a questionnaire on demographic information, cultural values along the vertical-horizontal, individualism-collectivism typology, and a brief measure of perceived positive and negative affect (PANAS Scales^52^). In total, the data collection took between 20–30 min. The identical design was used both Study 1 and Study 2.

## Results

We first present the results of our analyses looking at the impact of the induced emotions on changes participants’ upper-body posture. The results are presented separately for each study to demonstrate that the pattern of results obtained in Study 2 was similar to Study 1. We then present the results of the analyses investigating the impact of self-reported cultural values on posture using the large sample comprising both study samples. In the following, we report *p*-values for our main model comparisons, and we quantify the nature of statistical effects through reporting effect sizes, i.e., differences between group means, unstandardized *β*-coefficients, along with 95% confidence following prior recommendations^54^.

### Variation in upper-body postural expression of emotions (model 1 for both study 1 and 2)

**Figure 3:**
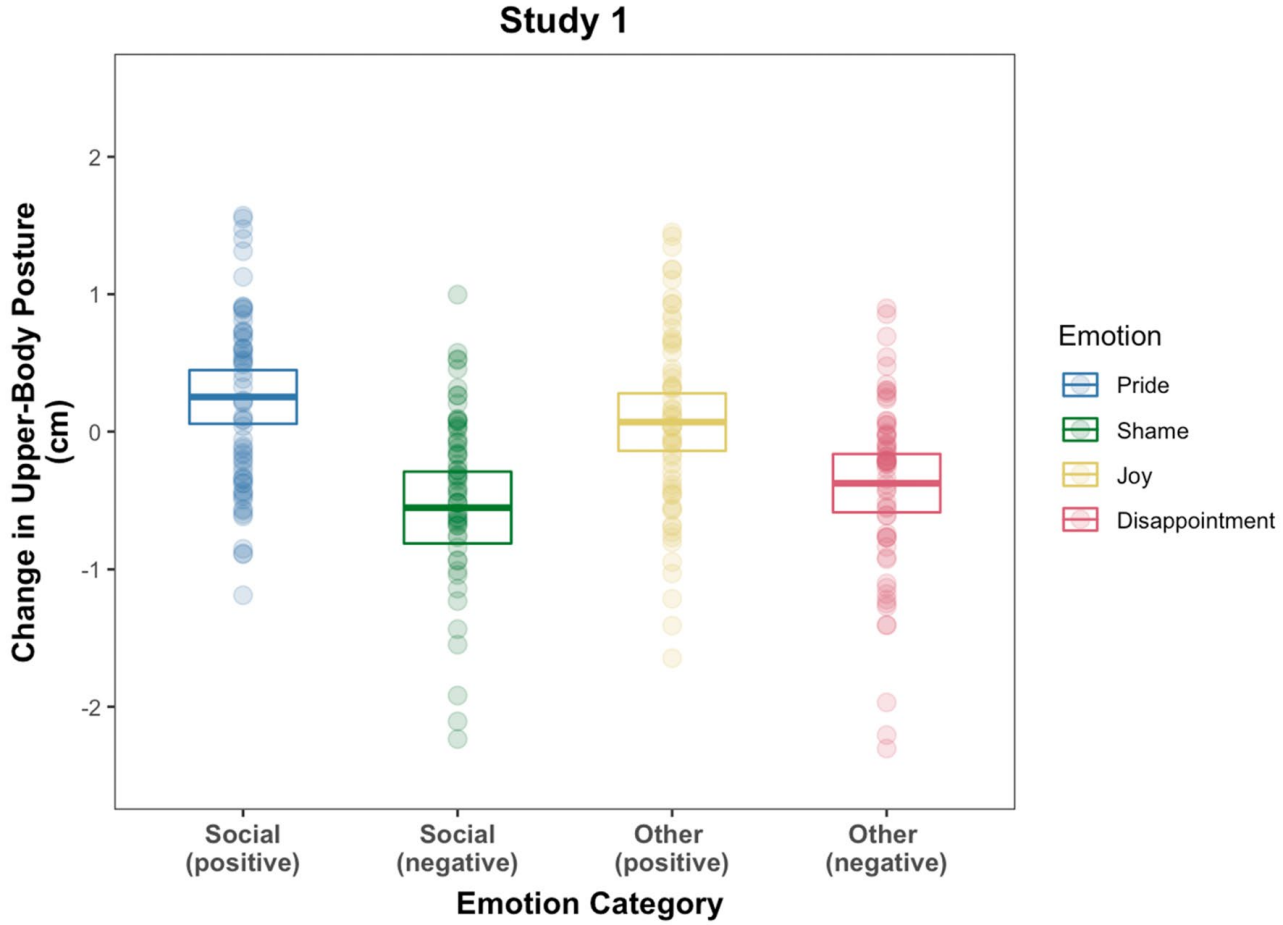
Results of Study 1. The average baseline-corrected change in upper-body posture. The center line of each box represents the group mean whereas the edges of each box represent the 95% confidence interval from the mean. Points represent each individual subject’s change in posture in the respective condition. Note that 10 values, either greater than 2.5 cm or smaller than  − 2.5 cm are not displayed to increase the scaling of the boxes but these values were included in the statistical analyses.

**Figure 4:**
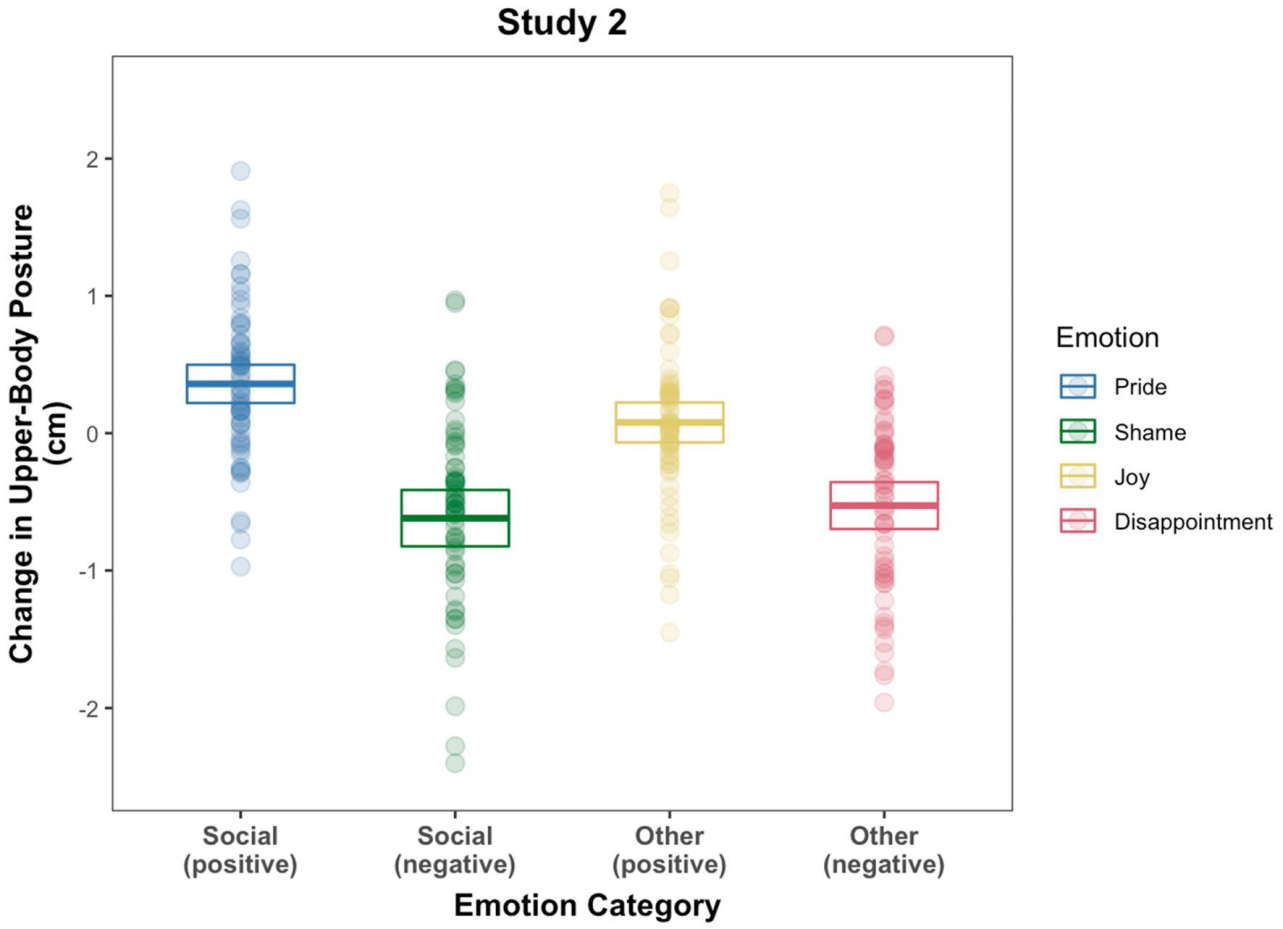
Results of Study 2. The average baseline-corrected change *in* upper-body posture. The center line of each box represents the group mean whereas the edges of each box represent the 95% confidence interval from the mean. Points represent each individual subject’s change in posture in the respective condition. Note that 10 values, either greater than 2.5 cm or smaller than  − 2.5 cm are not displayed to increase the scaling of the boxes but these values were included in the statistical analyses.

We first analyzed the data from each study separately to investigate the main effect of emotion on participants’ postural expression. We found that participants’ change in upper-body posture was systematically different between the four emotions, Study 1: *χ*^2^ (*df* = 6) = 62.34, *p* < 0.001, *R*^*2*^(marginal) = 0.05 (see Fig. 3; see also Table 1 for details) and Study 2: *χ*^2^ (*df* = 6) = 105.68, *p* < 0.001, *R*^*2*^(marginal) = 0.08 (see Fig. 4 see also Table 1 for details). The pattern of results was similar across both studies. More specifically, postural elevation was greatest when subjects recalled an event of feeling pride (Study 1: *M* = 0.25 cm, *SD* = 0.78 cm; Study 2: *M* = 0.36 cm, *SD* = 0.56 cm) compared to joy (Study 1: *M* = 0.07 cm, *SD* = 0.84 cm; Study 2: *M* = 0.08 cm, *SD* = 0.96 cm), disappointment (Study 1: *M* =  − 0.37 cm, *SD* = 0.85 cm; Study 2: *M* = -0.53 cm, *SD* = 0.69 cm), and lowest when recalling an experience of shame (Study 1: *M* =  − 0.55 cm, *SD* = 1.04 cm; Study 2: *M* =  − 0.62 cm, *SD* = 0.83 cm). We found no effect for gender (Study 1: *χ*^2^ (*df* = 4) = 7.52, *p* = 0.11; Study 2: *χ*^2^ (*df* = 4) = 3.19, *p* = 0.53), no interaction effect of gender and emotion (Study 1: *χ*^2^ (*df* = 3) = 6.99, *p* = 0.07; Study 2: *χ*^2^ (*df* = 3) = 1.51, *p* = 0.68), and no effect of trial (Study 1: *χ*^2^ (*df* = 1) = 0.25, *p* = 0.62; Study 2: *χ*^2^ (*df* = 1) = 1.79, *p* = 0.18). For Study 1, we found no main effect of the control predictor variable time-distance (*χ*^2^ (*df* = 1) = 3.75, *p* = 0.05) whereas for Study 2 the main effect was statistically significant (*χ*^2^ (*df* = 1) = 7.82, *p* = 0.01) but independent of the main effect of emotion.

**Table 1:** A summary of the average changes in upper-body posture elevation for each the four emotions in each of the studies.

	Pride	Joy	Shame	Disappointment
Study 1	*M* = 0.25, *SD* = 0.78, 95% CI [0.06 0.45]	*M* = 0.07, *SD* = 0.84, 95% CI [− 0.14 0.28]	*M* = − 0.37, *SD* = 0.85, 95% CI [− 0.59 − 0.17]	*M* = − 0.55, *SD* = 1.04, 95% CI [− 0.81 − 0.29]
Study 2	*M* = 0.36, *SD* = 0.56, 95% CI [0.21 0.5]	*M* = 0.08, *SD* = 0.59, 95% CI [− 0.07 0.25]	*M* = − 0.53, *SD* = 0.69, 95% CI [− 0.72 − 0.37]	*M* = − 0.62, *SD* = 0.83, 95% CI [− 0.82 − 0.39]

### Variation in lower-body postural expression of emotions (Model 2 for both Study 1 & 2)

In Study 1, the analyses of changes in participants’ lower-body posture (i.e., hip height) revealed a main effect of emotion, *χ*^2^ (*df* = 6) = 15.01, *p* = 0.02. However, we did not find the same pattern compared to the results on participants’ upper-body posture changes. With regards to lower-body posture, the change was greatest when subjects recalled feeling joy (*M* = 0.32, *SD* = 2.11) compared to shame (*M* = 0.14, *SD* = 1.78), disappointment (*M* = 0, *SD* = 0.69), and pride (*M* =  − 0.01, *SD* = 0.96). We found no effect for gender (*χ*^2^ (*df* = 4) = 7.52, *p* = 0.11), an interaction effect of gender and emotion (*χ*^2^ (*df* = 3) = 11.75, *p* = 0.01), no effect of trial (*χ*^2^ (*df* = 1) = 1.91, *p* = 0.17), and no effect of time-distance (*χ*^2^ (*df* = 1) = 0.02, *p* = 0.9).

In Study 2, the analyses of the change in participant’s lower-body posture revealed no main effect of emotion, *χ*^2^ (*df* = 6) = 5.52, *p* = 0.48. With regards to hip height, the change was greatest when subjects recalled feeling joy (*M* = 0.1, *SD* = 0.66) compared to shame (*M* = 0.02, *SD* = 0.65), disappointment (*M* =  − 0.01, *SD* = 0.58), and pride (*M* =  − 0.13, *SD* = 0.92). We found no effect for gender (*χ*^2^ (*df* = 4) = 3.19, *p* = 0.53), no interaction effect of gender and emotion (*χ*^2^ (*df* = 3) = 1.51, *p* = 0.68), no effect of trial (*χ*^2^ (*df* = 1) = 1.79, *p* = 0.18), and an effect of time-distance (*χ*^2^ (*df* = 1) = 7.82, *p* = 0.01).

### The influence of cultural values on the upper-body postural expression of emotions (Model 3 including study 1 & 2)

**Figure 5:**
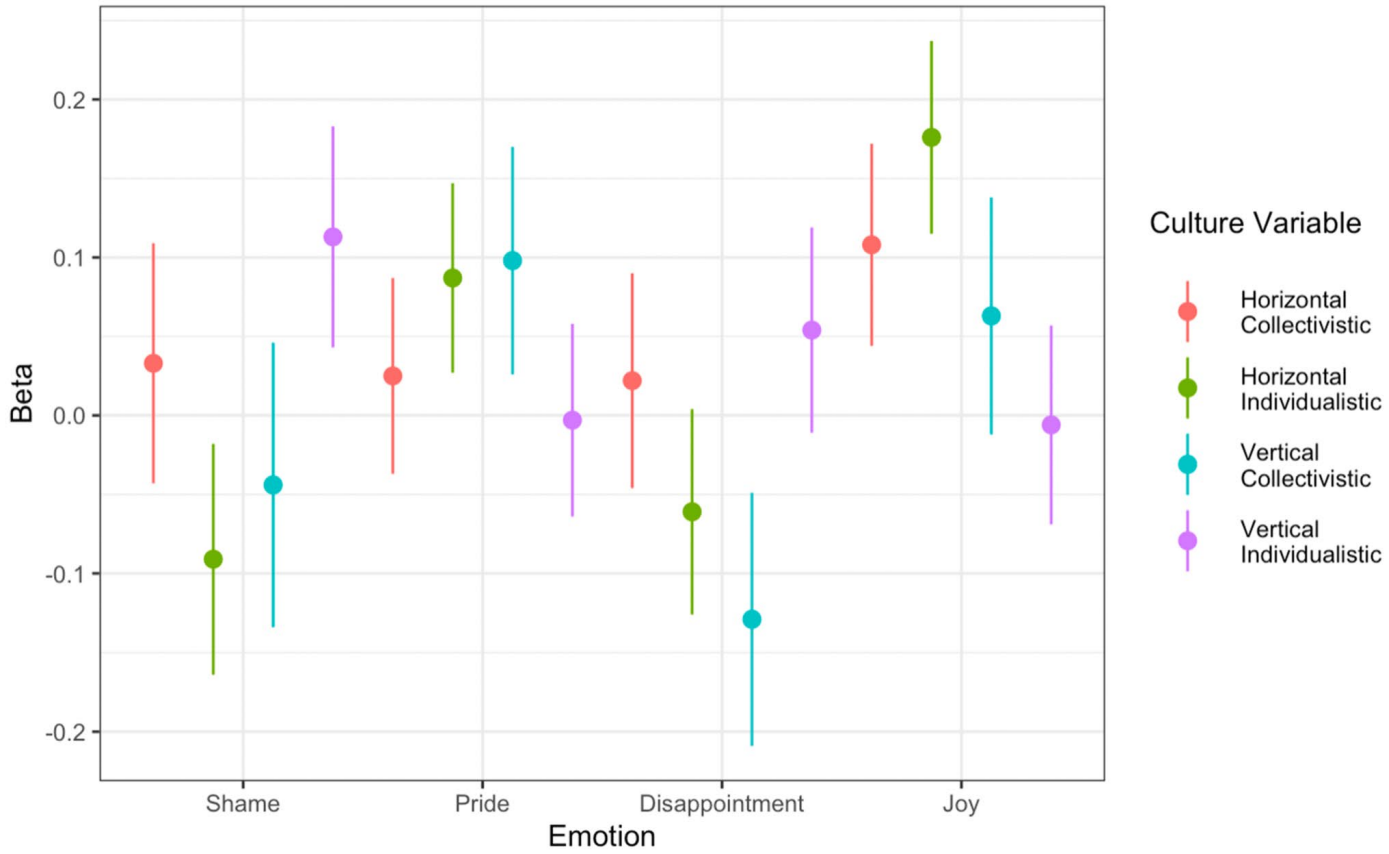
The influence of self-reported culture variables on participants’ upper-body posture for Studies 1 and 2 combined. The individual data points represent the beta-coefficients from fitting Model 3. The error bars represent + /− 1 standard error.

The cultural variables had a combined influence on participants’ change in upper-body posture, *χ*^2^ (*df* = 16) = 38.72, *p* < 0.001, *R*^*2*^(marginal) = 0.07 (see Fig. 5 and Table 2). We found an effect of gender (*χ*^2^ (*df* = 1) = 5.09, *p* = 0.02), no effect of trial (*χ*^2^ (*df* = 1) = 1.88, *p* = 0.17), an effect of time-distance (*χ*^2^ (*df* = 1) = 18.01, *p* < 0.001), and no effect of age (*χ*^2^ (*df* = 1) = 0.12, *p* = 0.73).

**Table 2:** The results of the analyses based on the aggregated data from studies 1 and 2.

Emotion	Category	Valence	Culture variable	*β-*coefficient	Standard error	95% CI (lower)	95% CI (upper)
Disappointment	Other	Negative	HoInd	− 0.061	0.065	− 0.190	0.068
Disappointment	Other	Negative	VeCol	− 0.129	0.080	− 0.287	0.029
Disappointment	Other	Negative	HoCol	0.022	0.068	− 0.113	0.157
Disappointment	Other	Negative	VeInd	0.054	0.065	− 0.075	0.183
Shame	Social	Negative	HoInd	− 0.091	0.073	− 0.235	0.053
Shame	Social	Negative	VeCol	− 0.044	0.090	− 0.222	0.134
Shame	Social	Negative	HoCol	0.033	0.076	− 0.117	0.183
Shame	Social	Negative	VeInd	0.113	0.070	− 0.026	0.252
Joy	Other	Positive	HoInd	0.176	0.061	0.055	0.297
Joy	Other	Positive	VeCol	0.063	0.075	− 0.085	0.211
Joy	Other	Positive	HoCol	0.108	0.064	− 0.019	0.235
Joy	Other	Positive	VeInd	− 0.006	0.063	− 0.131	0.119
Pride	Social	Positive	HoInd	0.087	0.060	− 0.032	0.206
Pride	Social	Positive	VeCol	0.098	0.072	− 0.044	0.240
Pride	Social	Positive	HoCol	0.025	0.062	− 0.098	0.148
Pride	Social	Positive	VeInd	− 0.003	0.061	− 0.124	0.118

*HoCol* horizontal-collectivism, *HoInd* horizontal-individualism, *VeCol* vertical-collectivism, *VeInd* vertical-individualism.

### Exploratory analyses

Exploratory analyses are reported in the online supplement. We analyzed whether the absolute difference in postural expression between the positive (pride) and negative (shame) social emotion can be explained by the general expected difference between positive and negative emotions. Additional exploratory analyses regarding associations between changes in participants’ upper-body posture and their PANAS-ratings of the extent to which they have felt positive and negative feelings and emotions in everyday life can be found in the online supplement.

## Discussion

Previous research had not directly investigated the impact of cultural values on the experience and expression of self-evaluative social emotions, in particular when non-verbal measures are used. The present set of studies are the first to capture participants’ live postural expression after they recalled emotion episodes reflecting shame, pride, disappointment, and joy. We found that positive emotions elevated participants’ upper-body posture whereas negative emotions resulted in lowered upper-body posture. This pattern was found across two separate samples and, by and large, replicates prior work demonstrating that positive emotions have an uplifting effect on body posture whereas negative emotions do not^23,38,45^. The automated assessment of adults’ body posture thus provides an objective means to measure degrees of valence that distinguish positive (high valence) from negative (low valence) emotions^51^. In addition, our results showed that the difference between positively and negatively valenced emotions was greater for social than for more basic emotions. Specifically, when participants recalled a pride episode this resulted in greater postural elevation than recalling joy. Likewise, recalling shame resulted in a more slouched upper-body posture than recalling disappointment. This pattern of results speaks to previous research arguing that social emotions are deep-felt by individuals^55^. Social emotions are dependent on other people^22^ and, thus, are key components in the regulation of social behavior^56^. Shame, for example, is felt as a penalty for violating (social) norms or failing to meet (social) standards. In contrast, pride is an emotion experience to meeting and exceeding (social) standards^57^. In addition, both shame and pride are influenced by the presence of observers such that their expressions are exaggerated when they are being watched. The fact that participants in the current study were observed by the experimenter may have further contributed to the greater expressiveness of the social compared to the basic emotions.

Overall, our findings provide further support for conceptualizing social emotions as commitment devices to regulate social relationships^12,55,58^. On such an account, social, self-evaluative, emotions should be particularly influenced by cultural values given that culture uniquely shapes societally norms and values^57,59^. Cross-cultural work shows that cultures, e.g., Western individualistic compared to Eastern collectivist, impact the degree to which social emotions such as shame are experienced and expected in everyday life^56^. Furthermore, while the expression and prevalence of social emotions may vary across cultures the general structure of the expression of social emotions, e.g., in the case of pride, appears to be a cross-cultural universal^60^. Against this background, the results of the present studies suggest that the influence of cultural values may be more nuanced than previously documented. Our systematic assessment of individualism and collectivism along horizontal and vertical dimensions revealed that the postural expression of social emotions was uniquely impacted by these self-reported cultural values. In the case of pride, an interesting finding was that horizontal collectivistic values had a posture-elevating effect on participants’ upper-body posture. Pride is an emotion geared toward enhancing and affirming the self (p. 273)^61^. In part, the emotional experience of pride involves judging one’s own abilities against others which seems to contradict cultural values of equality and interdependence of the self (*horizontal collectivism*), in particular when pride is about one’s individual self^61^. Indeed, it has been found that in some collectivistic cultures pride is not a desirable emotion despite its positive valence^62^. To further the understanding of the complexities concerning cultural variation in pride expression it will be important for future research to distinguish between group pride (i.e., pride that results from accomplishments of others) and pride that results from one’s own accomplishments in order. Alternatively, the effect of the cultural pattern of horizontal collectivism on the postural expression of pride may be because individuals view themselves in the same way as they view others within their own group, including their emotions. Research documenting an in-group bias in both emotion recognition and emotion expression may speak to this assumption^61^.

A different pattern was found for the influence of the four cultural variables on the postural expression of the emotion joy. Here the scale *horizontal individualistic* had the greatest influence while the scale *vertical individualistic* resulted in a negative beta-slope on the upper-body expression after adults recalled experiencing joy. Horizontal individualism is characterized by an independent self which—at the same time—is equal in status with others^16^. Vertical individualism further emphasizes an independent self, but people do not see each other as the same, thus, accepting inequality^16^. The finding of differences in the effect of these two cultural patterns on the postural expression of joy (both in direction and in strength) speaks to previous research arguing that there are cultural nuances in the experience and expression of emotions, even between two cultural patterns that share similar attributes (such as that both horizontal individualism and vertical individualisms postulate an autonomous self)^61^.

In comparison to positive emotions, the impact of cultural values on negative emotions was more bidirectional: *Horizontal collectivistic* and *vertical individualistic* scales had an elevating effect on posture whereas *horizontal individualistic* and *vertical collectivistic* scales had a negative effect. Vertical collectivism views the self as interdependent (similar to horizontal collectivism) but the individual self is different from the self of others, reflecting inequality (similar to vertical individualism)^16^. Overall, the effects of cultural values on posture were similar across the negative compared to the positive emotions. One possible explanation for this pattern is the negativity bias in emotion perception and expression which has deep ontogenetic roots^63,64^. Negative emotions signal danger and prompt rapid responses in observers to adjust their behavior. This biological adaption may overwrite the cultural influences on negative emotions, but this needs to be a topic for future research.

## Limitations

The present studies did not systematically investigate the effects of demand characteristics on changes in participants’ body posture. In our studies, the experimenter providing the instructions was turned away from the situation while participants walked toward the Kinect while the crucial measure of body posture was taken during each trial. It is, however, plausible that participants expressed each emotion in a way they thought was the most appropriate (or most expected of them). In addition, individuals with a certain value orientation may have been better at displaying emotions in line with (perceived) expectations by the experimenter. In this way participants may have expressed a ‘cultural prototype’ or a display rule for each emotion as opposed to expressing their own genuine feelings at the moment when we took the posture measurement. In addition, an as is the case with the majority of studies with adults, participants were aware of being recorded. It would require a different study design, one that involved temporarily deceiving participants to motivate them to behave ‘as if no one was watching’. We did not think that such levels of deceit were justified given our main research questions. The possibility that demand characteristic influenced participants’ body posture does not undermine or confound our experimental manipulation or individual differences analyses. It does however provide an interesting avenue for future research, one that tests whether reducing demand characteristics results in different patterns compared to the ones we found. Although social emotions necessarily depend on other people, at the time the emotion is experienced the physical presence of another person is not required^22,50^. In other words, social emotions are experienced regardless of whether another person is present or not. We consider this is to be an empirical question in its own right.

While our findings, together with previous research on cultural similarities and differences in emotion expression^24,25^, may lead to new hypotheses regarding cultural influences on the emotions, it is important to note, that the pattern of results observed in the present set of studies is specific to the emotions that we selected, the instructions for recalling the emotions, and the sample of participants we recruited (undergraduate students enrolled in an international university that has a distinctive focus on intercultural understanding). It will therefore be important to expand the range of social emotions and to compare social emotions of the same valence (for example, two negative social emotions, such as shame and guilt). It will also be important to include a different sample of participants (other than undergraduate college students; recruited from different cultural contexts) and to observe postural expression in naturalistic situation when emotions are expressed spontaneously. In addition, the cultural values of interest were chosen to reflect the established independence-interdependence spectrum to understand how cultural values shape the expression of self-evaluative social emotions. Building on these initial findings, further (and more specific) values can be investigated with regard to their influence on emotions. Previous research on values suggests that, for example, the specific motivational element of a value in conjunction with the value’s focus (social versus personal) shape how individuals cope with their environment^11^. Thus, a value-based account of emotions may help disentangle cultural influences in the emotion process^11,65^.

## Conclusions

Taken together, the results of the present studies suggest that cultural values along the vertical-horizontal and individualism-collectivism typology shape the postural expression of self-evaluative social emotions. Importantly, instead of assessing differences in cultural values between groups, we assessed each participant’s cultural values along this typology, thus, recognizing that specific cultural values may coexist within individuals^15^. This investigation yielded initial evidence of the nuances in the way cultural values influence the postural expression of emotions. Cultural values viewed as subjective construal rather than dichotomizing individualistic or collectivistic group patterns can explain individual differences in the function of body posture for the experience and expression of social emotions. Framed this way, the field may overcome “value judgment that one cultural system (often individualism) was better or more advanced than the other cultural system (often collectivism)” (p. 111)^66^ and contribute new insights into the ingredients for any observed differences and the subtle ways that cultural values exert influence on cognition and behavior.

## Methods

The study protocol complies with all ethical regulations for research with human subjects and was approved by the Institutional Review Board of New York University Abu Dhabi (UAE) prior to data collection (IRB Protocol # 028-2018). Written informed consent for study participation was obtained from all subjects prior to participating. At the end of the study, all participants were debriefed and received monetary compensation equivalent to 7 USD for their participation.

## Participants study1

Students (*n* = 66, 36 females) were on average 20.11 years old (*SD* = 1.46). Students were from diverse cultural backgrounds, 15 classified themselves as East-Asian, 9 each as South-Asian, European and African, 5 as American, and 4 as Arabic (missing information for 15 students). They lived, on average, 14.93 years in their country of origin (*SD* = 5.87). Students were asked to rate how much they identify with the cultural beliefs, values and norms of their country of origin (*M* = 3.44, *SD* = 0.90, using a 5-point Likert-type scale [1 = *not at all*; 3 = *somewhat*; 5 = *very much*]). Half of the students (34) were in their freshmen year, 13 in the sophomore year, 6 in the junior year, and 13 in their last year of college. Students were enrolled in 18 different majors (out of the total of 24 undergraduate majors offered at the university). Students (83%) who knew their GPA reported an average GPA of 3.67 (*SD* = 0.31). The remaining students gave an estimate of their GPA (*M* = 3.38, *SD* = 0.36). Two of the 66 subjects were excluded because they did not provide data on every test trial due to technical equipment failure.

## Participants study 2

Students (*n* = 66, 31 females) were, on average, 19.55 years (*SD* = 1.60). The cultural background of students was again diverse; 17 students classified themselves as from a European background, 14 students as East-Asian, 11 students as South-Asian, 10 as African, 7 as American, and 3 as Arabic (missing information for 4 students). They lived, on average, 14.51 years in their country of origin (*SD* = 6.11). When asked how much they identify with the cultural beliefs, values and norms of their country of origin, the mean was very similar to the first sample (*M* = 3.45, *SD* = 0.88). Almost half of the students (*n* = 32) were in their freshmen year, 11 were sophomores, 7 juniors, and 16 seniors. Students were enrolled in 16 different majors. Forty-one percent of students knew their GPA (*M* = 3.69, *SD* = 0.26); 42% reported an estimate of their GPA (*M* = 3.50, *SD* = 0.30). The information was missing for 17%. One of the 66 subjects was excluded because no data could be recorded for any of the test trials due to technical equipment failure.

## Procedure and measures

The data was collected by the first author. Participants were met in the study room. They were briefed about the aims and procedure of the study and gave written informed consent prior to participating. At the beginning of the data collection, participants were given the following instruction: “This is a method to measure emotional expression. For this purpose, we would like you to walk toward the Kinect twice for each emotion. At the very beginning, we will conduct a baseline measurement for which we ask you to walk in a relaxed natural manner (again twice). Afterward I will read out the instructions for each emotion, four in total. In total, we will take 10 measurements. We record only postural information. Are you ready for the baseline walk? Please walk in a relaxed natural manner.” Following the two baseline trials, participants were asked to recall an emotional experience. Each emotion trial began with the experimenter providing participants with emotion induction instructions (see Table 3).

**Table 3:** Emotion induction instructions.

Emotion	Induction instructions
Joy (positive, other)	The emotion to be displayed is joy. Can you recall an event that made you feel joy? It is important that you think of a situation that is not related to pride. Try to recall that feeling. Imagine s specific situation and the surroundings. Take your time until you feel the emotion. Once you are ready you can walk toward the camera
Pride (positive, social)	The emotion to be displayed is pride. Can you recall an event that made you feel pride? Try to recall that feeling. Imagine a specific situation and the surroundings. Take your time until you feel the emotion. Once you are ready you can walk toward the camera
Disappointment (negative, other)	The emotion to be displayed is disappointment. Can you recall an event that made you feel disappointment? It is important that you think of a situation that is not related to shame. Try to recall that feeling. Imagine a specific situation and the surroundings. Take your time until you feel the emotion. Once you are ready you can walk toward the camera
Shame (negative, social)	The emotion to be displayed is shame. Can you recall an event that made you feel shame? Try to recall that feeling. Imagine a specific situation and the surroundings. Take your time until you feel the emotion. Once you are ready you can walk toward the camera

Per emotion we captured two successive walks. All walks were initiated at about 7 m away from the Kinect camera. The Kinect camera was placed at height = 0.85 m from the ground and at distance from participants’ starting position = 3.7 m, with an angle of the camera = 11°. To minimize a potential bias due to the presence of the experimenter in the study room who was aware of the hypotheses, the experimenter turned her back to the participant during the walks so as to not face nor look at participants. For each walk, the recording was manually initiated by the experimenter once subjects started their walk and was manually stopped by the experimenter once subjects finished their walk. This was done via the live camera feed shown on the laptop. Only the participant and the experimenter were present during the recording. Following the two walks for each emotion, participants briefly described the event they recalled on a paper before continuing with the next emotion induction. Participants could sit at a table that was placed in the center of the room while completing the document. We scanned each document which contained description of the events participants recalled during each emotion induction. We did not qualitatively analyze these vignettes but the data are available from the authors upon request.

### Cultural values along vertical-horizontal, individualism-collectivism typology

We used the Individualism-Collectivism Scale^67^ to assess four dimensions of collectivism and individualism: vertical collectivism, vertical individualism, horizontal collectivism, and horizontal individualism. Each dimension was assessed by four items, answered on a 9-point Likert-type scale (ranging from 1 = never or definitely no to 9 = always or definitely yes). Though participants used the entire scale range for their responses, an inspection of the frequency of values at the item level indicated that responses were skewed toward the upper end of the scale (means ranged between 4.29 to 7.65; skewness ranged between  − 1.43 to 0.11).

We created latent constructs for each of the four dimensions, using confirmatory factor analysis (CFA) for the combined sample of both Study 1 and Study 2. The analyses were run in MPlus (Version 7.4 Mac) using Maximum Likelihood Robust (MLR) estimator. The initial CFA indicated poor model fit (RMSEA = 0.085 [90% CI: 0.067–0.103]; CFI = 0.771; TLI = 0.719; SRMR = 0.094). A closer inspection of the results revealed that for each dimension, the loading of one item was not significant (vertical collectivism: item 15 [*It is important to me that I respect the decisions made by my groups.*]; vertical individualism: item 16 [*When another person does better than I do, I get tense and aroused.*]; horizontal collectivism: item 3 [*If a classmate gets a prize, I would feel proud.*]; horizontal individualism: item 14 [*My personal identity, independent of others, is very important to me.*]). The CFA was repeated without these four items which increased fit indices (RMSEA = 0.063 [90% CI: 0.030–0.090]; CFI = 0.922; TLI = 0.892; SRMR = 0.064). We inspected modification indices based on which we allowed two items to correlate (item 5 [*Winning is everything.*] WITH item 9 [*Family members should stick together, no matter what sacrifices are required.*]). The resulting model fit was good (RMSEA = 0.049 [90% CI: 0.000–0.080]; CFI = 0.953; TLI = 0.934; SRMR = 0.065). The factor loadings of all items were significant (*p* = 0.000) and the standardized factor loadings of all but one item were above 0.500 (see Table 4). The reliability of each of the four dimensions was acceptable in the present data set (Cronbach’s alpha ranged between 0.62 and 0.76 which is comparable to the reliability reported in previous research^68^). We extracted the factor scores for each of the four cultural dimensions for each participant to explore whether change in postural expression is influenced by participants’ cultural values.

**Table 4:** Standardized factor loadings.

Dimension	Item	Estimate	S.E
Vertical collectivism	Parents and children must stay together as much as possible	0.570	0.107
	It is my duty to take care of my family, even when I have to sacrifice what I want	0.603	0.099
	Family members should stick together, no matter what sacrifices are required	0.941	0.095
Vertical individualism	It is important that I do my job better than others	0.504	0.079
	Winning is everything	0.828	0.077
	Competition is the law of nature	0.769	0.064
Horizontal collectivism	The well-being of my classmates is important to me	0.391	0.109
	To me, pleasure is spending time with others	0.529	0.096
	I feel good, when I cooperate with others	0.957	0.103
Horizontal individualism	I’d rather depend on myself than others	0.721	0.090
	I rely on myself most of the time; I rarely rely on others	0.712	0.101
	I often do “my own thing”	0.552	0.105

All estimates are statistically significant (*p* < 0.01).

### Positive and negative affect

The PANAS (Positive and Negative Affect Schedule) Scales^52^ were used as a brief measure of individuals’ perceived positive and negative feelings and emotions in everyday life. Previous research has documented that positive and negative affect are two dominant and relatively independent dimensions of affect^52^. Participants are instructed to indicate the extent to which they have felt different feelings and emotions over the past week, using 5-point Likert-type scale (ranging from 1 = very slightly or not at all to 5 = very much). Each dimension is measured with 10 items. We created latent constructs for each of the four dimensions, using confirmatory factor analysis (CFA) for the combined sample of both Study 1 and Study 2. The analyses were run in MPlus (Version 7.4 Mac) using Maximum Likelihood Robust (MLR) estimator. The initial CFA indicated less optimal model fit (RMSEA = 0.056 [90% CI: 0.039–0.072]; CFI = 0.897; TLI = 0.884; SRMR = 0.080). A closer inspection of the results revealed that the factor loading of item 12 (“alert”) was not significant. The CFA was repeated without item 12 which increased fit indices (RMSEA = 0.029 [90% CI: 0.000–0.052]; CFI = 0.975; TLI = 0.971; SRMR = 0.067). The reliability of the two dimensions was acceptable in the present data (Cronbach’s alpha was 0.86 for positive affect and 0.81 for negative affect which is comparable to the reliability reported in previous research^52^). We extracted the factor scores for each of the two dimensions for each participant to explore whether changes in participants’ upper-body posture correlated with their PANAS-ratings of the extent to which they have felt positive and negative feelings and emotions in everyday life.

## Statistical analyses

The posture data were recorded and pre-processed using pre-established algorithms (Github-link: https://github.com/rhepach/Kinect). For each test trial, we extracted 20 data points per participants reflecting the xyz-coordinates of each of the skeletal joints. The 20 data points resulted from dividing up the distance that participants walked toward the Kinect into 20 equally sized distance bins between 3.2 and 1.2 m away from the Kinect (see also^47,49^). This resulted in 20 baseline-corrected data points reflecting participants’ walk toward the Kinect motion sensor. For each of the 20 data points (bins across subjects) from the Kinect camera we determined the proportion of found samples across all participants and trials. Those bins for which the proportion was below 90% of the median proportion were excluded from the statistical analyses (details are provided in the analysis scripts: https://osf.io/v56jn/. This final validation check revealed that the first 16 bins (18 bins for Study 2), from 3.2 to 1.6 m, provided sufficient data density. Therefore, we entered 16 (18 Study 2) data points per trial per participant which we here refer to as the predictor variable ‘time-distance’ because it reflects the combined information from participants walking toward the Kinect during each test trial.

For the change in upper-body posture (i.e., the change in chest height) we ran linear mixed models (Gaussian error distribution) in *R* using the function *lmer()* (package *lme4*)^69^. We ran the same models for the change in lower-body posture (i.e., the change in hip height) as control analyses. The statistical significance was tested through calculating likelihood-ratio tests using the function *drop1()*. In each of the following models we included trial (4 data points) and time-distance of participants walking toward the Kinect-camera (16 data points, 18 data points for Study 2, per subject and trial) as control variables, subject as a random intercept, random slopes of time-distance on subject, random slopes of trial on subject, and random slopes for emotion on subject.

All our analyses scripts as well as the data recorded with the Kinect are provided here: https://osf.io/v56jn/. We preregistered our analyses for Study 1 (https://osf.io/9qbpw) and for Study 2 (https://osf.io/4cjga). The following changes were made to the preregistered models to make the models more parsimonious and to address over-fitting: (1) We did not model time-distance to interact with the predictor variables gender, emotion, and culture. This reduced the complexity of the statistical model and, in addition, initial analyses indicated no interaction effects including time-distance. (2) We did not include culture as a fixed effect in the separate models for each culture but rather included this variable in our combined analysis of both Study 1 and 2 with greater statistical power. This is also a result of realizing cultural variation cannot be expressed in a single variable but that a more accurate approach is to include the four cultural variables within the same model: Horizontal-Collectivism, Horizontal-Individualism, Vertical-Collectivism, Vertical-Individualism. As a consequence, we ran a total of five analyses. They are detailed in the following. The models 1 and 2 were fitted for the samples of Study 1 and 2 separately.

### Change in upper-body posture (model 1)

The dependent measure was the change in participants’ upper-body posture (baseline-corrected change in chest height). The predictor variables were emotion (shame, pride, joy, disappointment) and gender. We modelled both main effects as well as an interaction of emotion and gender. The *R*-code for this model was:

$$ {\text{lmer}}({\text{PostureChange }}\sim {\text{ TimeDistance }} + ~{\text{Emotion}}*{\text{Gender }} + {\text{ Trial}} + {\text{ }}\left( {{\text{1}} + {\text{ TimeDistance}}\left( {{\text{z}} - {\text{transformed}}} \right){\text{ }} + {\text{ Trial }}||{\text{ Subject}}} \right){\text{ }} + {\text{ }}\left( {0 + {\text{Emotion}}|{\text{ Subject}}} \right) $$

The preregistered analysis was:

$$ {\text{lmer}}({\text{PostureChange}}~({\text{TimeDistance}} + {\text{Emotion}} + {\text{Gender}} + {\text{CulturalDimension}})^{2}  + {\text{Trial}} + (1 + {\text{TimeDistance}}({\text{z}} - {\text{transformed}}) + {\text{Trial}}||{\text{Subject}}) + (0 + {\text{Emotion}}|{\text{Subject}}) $$

We replaced the following two analyses with Model 1 above:

$$ {\text{lmer}}({\text{PostureChange~}}({\text{TimeDistance}} + {\text{Emotion}} + {\text{Gender}} + {\text{CulturalDimension}})^{3}  + {\text{Trial}} + (1 + {\text{TimeDistance}}({\text{z}} - {\text{transformed}}) + {\text{Trial}}||{\text{Subject}}) + (0 + {\text{Emotion}}|{\text{Subject}}) $$

and

$$ {\text{lmer}}({\text{PostureChange }}\sim {\text{ TimeDistance}}*{\text{Emotion}}*{\text{Gender}}*{\text{CulturalDimension}} + {\text{ Trial}} + {\text{ }}\left( {{\text{1}} + {\text{ TimeDistance}}\left( {{\text{z}} - {\text{transformed}}} \right){\text{ }} + {\text{ Trial }}||{\text{ Subject}}} \right){\text{ }} + {\text{ }}\left( {0 + {\text{Emotion}}|{\text{ Subject}}} \right) $$

### Change in lower-body posture (model 2)

The dependent measure was the change in participants’ lower-body posture (baseline-corrected change in hip height). The predictor variables were emotion (shame, pride, joy, disappointment) and gender. We modelled both main effects as well as an interaction of emotion and gender. The model structure was identical to Model 1. In addition, the preregistered analyses with hip height as the dependent measure were identical to those noted above.

### Influence of participants’ cultural values on changes in upper-body posture (model 3)

As noted above, this model was not preregistered but rather tests the effect of cultural values with both samples combined instead of for each sample separately. To investigate the influence of cultural identity on participants’ upper-body posture, we combined the sample of both studies. The dependent measure was the change in participants’ upper-body posture (baseline-corrected change in chest height). We included as predictor variables the four interactions of emotion (shame, pride, joy, disappointment) with each of the scaled cultural variables (Horizontal-Collectivism [HoCol], Horizontal-Individualism [HoInd], Vertical-Collectivism [VeCol], Vertical-Individualism [VeInd]), time-distance (scaled), trial, gender, and participants’ age (scaled). The random effects structure was identical to models 1 and 2. We tested the combined influence of all culture variables by comparing the full model to a reduced model including only the main effects of emotion, time-distance, trial, gender, age, as well as all random effects. The model structure was as follows:

$$ {\text{bothStudies}}.{\text{model1 }} = {\text{ lmer}}({\text{PostureChange }}\sim {\text{ TimeDistance}}\left( {{\text{z}} - {\text{transformed}}} \right){\text{ }} + ~{\text{Emotion}}*{\text{VeCol }} + {\text{Emotion}}*{\text{HoInd }} + {\text{ Emotion}}*{\text{HoCol }} + {\text{ Emotion}}*{\text{VeInd }} + {\text{ Gender}}.{\text{x }} + {\text{ Trial}}.{\text{phase }} + {\text{ z}}.{\text{age }} + {\text{ }}\left( {{\text{1}} + {\text{ TimeDistance}}\left( {{\text{z}} - {\text{transformed}}} \right){\text{ }} + {\text{ num Trial phase }}||{\text{ Subject}}} \right){\text{ }} + {\text{ }}\left( {0 + {\text{Emotion}}|{\text{ Subject}}} \right) $$

## Power analyses

For each of the main analyses, we ran data simulations to calculate post-hoc statistical power for comparing the full model to the respective reduced models. Using the *R*-package simr^53^ we ran 1,000 iterations for each model comparison. The results indicated that we had sufficient statistical power for our main analyses on in Study 1 (Model 1), 1 − *β* = 0.98, for our main analyses in Study 2 (Model 1), 1 − *β* = 0.99, and for our main model investigating the effect of culture based on data from both studies (Model 3), 1 − *β* = 0.99.

## Supplementary Information

Supplementary Information.

## Data Availability

The data that support the findings of this study is openly available on OSF: https://osf.io/v56jn/.

## References

[1] Henrich J (2017). The secret of success: How culture is driving human evolution, domesticating our species, and making us smarter.

[2] Tomasello M (2018). A natural history of human thinking, Harvard.

[3] Velez-Agosto NM, Soto-Crespo JG, Vizcarrondo-Oppenheimer M, Vega-Molina S, Garcia Coll C (2017). Bronfenbrenner's bioecological theory revision: Moving culture from the macro to the micro. Perspect. Psychol. Sci..

[4] Han S, Northoff G (2008). Culture-sensitive neural substrates of human cognition: A transcultural neuroimaging approach. Nat. Rev. Neurosci..

[5] Kitayama S, Markus HR, Kurokawa M (2000). Culture, emotion, and well-being: Good feelings in Japan and the United States. Cogn. Emot..

[6] Nisbett RE, Masuda T (2003). Culture and point of view. Proc. Natl. Acad. Sci..

[7] Nisbett RE, Miyamoto Y (2005). The influence of culture: Holistic versus analytic perception. Trends Cogn. Sci..

[8] Park J, Kitayama S, Markus HR, Coe CL, Miyamoto Y, Karasawa M, Ryff CD (2013). Social status and anger expression: The cultural moderation hypothesis. Emotion.

[9] Markus HR, Kitayama S (1991). Culture and the self: Implications for cognition, emotion, and motivation. Psychol. Rev..

[10] Sortheix FM, Schwartz SH (2017). Values that underlie and undermine well-being: Variability across countries. Eur. J. Pers..

[11] Tamir M, Schwartz SH, Cieciuch J, Riediger M, Torres C, Scollon C, Vishkin A (2016). Desired emotions across cultures: A value-based acoount. J. Personal. Soc. Psychol..

[12] Van Kleef GA (2009). How emotions regulate social life: The Emotions as Social Information (EASI) model. Curr. Dir. Psychol. Sci..

[13] Van Kleef GA (2010). The emerging view of emotion as social information. Soc. Pers. Psychol. Compass.

[14] Darwish A-FE, Huber GL (2003). Individualis vs collectivism in different cultures: A cross-cultural study. Intercult. Educ..

[15] Oyserman D, Coon HM, Kemmelmeier M (2002). Rethinking individualism and collectivism: Evaluation of theoretical assumptions and meta-analyses. Psychol. Bull..

[16] Singelis TM, Triandis HC, Bhawuk DPS, Gelfand MJ (1995). Horizontal and vertical dimensions of individualism and collectivism: A theoretical and measurement refinement. Cross-Cult. Res..

[17] Triandis HC (1995). Individualism and collectivism.

[18] Chirkov V, Ryan RM, Kim Y, Kaplan U (2003). Differentiating autonomy from individualism and independence: A self-determination theory perspective on internalization of cultural orientations and well-being. J. Pers. Soc. Psychol..

[19] Frank R (1988). Passion with reason: The strategic role of the emotions.

[20] Van Kleef GA, Cheshin A, Fischer AH, Schneider IK (2016). Editorial: The social nature of emotions. Front. Psychol..

[21] Van Kleef GA (2017). The social effects of emotions are functionally equivalent across expressive modalities. Psychol. Inq..

[22] Hareli S, Parkinson B (2008). What's social about social emotions?. J. Theory Soc. Behav..

[23] Dael N, Mortillaro M, Scherer KR (2012). Emotion expression in body action and posture. Emotion.

[24] Matsumoto D, Hee Yoo S, Fontaine J, Anguas-Wong AM, Arriola M, Grossi E (2008). Mapping expressive differences around the world: The relationship between emotional display rules and individualism versus collectivism. J. Cross Cutl. Psychol..

[25] Safdar S, Friedlmeier W, Matsumoto D, Hee Yoo S, Kwantes CT, Kakai H (2009). Variations of emotional display rules within and across cultures: A comparison between Canada, USA, and Japan. Can. J. Behav. Sci..

[26] Uchida Y, Townsend SS, Markus HR, Bergsieker HB (2009). Emotions as within or between people? Cultural variation in lay theories of emotion expression and inferecne. Pers. Soc. Psychol. Bull..

[27] Brosch T, Pourtois G, Sander D (2010). The perception and categorization of emotional stimuli: A review. Cogn. Emot..

[28] Cowen AS, Laukka P, Elfenbein HA, Liu R, Keltner D (2019). The primacy of categories in the recognition of 12 emotions in speech prosody across two cultures. Nat. Human Behav..

[29] de Gelder B, de Borst AW, Watson R (2015). The perception of emotion in body expressions. Cogn. Sci..

[30] Martinez L, Falvello VB, Aviezer H, Todorov A (2016). Contributions of facial expressions and body language to the rapid perception of dynamic emotions. Cogn. Emot..

[31] Ekman P (1992). Facial expressions of emotions: New findings, new questions. Psychol. Sci..

[32] Ekman P (1993). Facial expression and emotion. Am. Psychol..

[33] Keltner D, Tracy J, Sauter DA, Cordaro DC, McNeil G (2016). Expression of emotions, in Handbook of emotions.

[34] Aviezer H, Trope Y, Todorov A (2012). Body cues, not facila expressions, discriminate between intense positive and negative emotions. Science.

[35] Aviezer H, Hassin RR, Ryan J, Grady C, Susskind J, Anderson A, Bentin S (2008). Angry, disgusted, or afraid? Studies on the malleability of emotion perception. Psychol. Sci..

[36] Borhani K, Ladavas E, Maier ME, Avenanti A, Bertini C (2015). Emotional and movement-related body postures modulate visual processing. Soc. Cogn. Affect. Neurosci..

[37] Filmer HL, Monsell S (2013). TMS to V1 spares discrimination of emotive relative to neutral body postures. Neuropsychologia.

[38] Montepare JM, Goldstein SB, Clausen A (1987). The identification of emotions from gait information. J. Nonverbal Behav..

[39] Lewis M, Alessandri SM, Sullivan ME (1992). Differences in shame and pride as a function of children's gender and task difficulty. Child Dev..

[40] Shiota MN, Campos B, Keltner D (2003). The faces of positive emotion: Prototype displays of awe, amusement, and pride. Ann. N. Y. Acad. Sci..

[41] Tracy JL, Robins RW (2004). Show your pride: Evidence for a discrete emotion expression. Psychol. Sci..

[42] Shariff AF, Tracy JL (2009). Knowing who's boss: Implicit perceptions of status from the nonverbal expression of pride. Emotion.

[43] Martens JP, Tracy JL (2013). The emotional origins of a social learning bias: Does the pride expression cue copying?. Soc. Psychol. Personal. Sci..

[44] Schwartz B, Tesser A, Powell E (1982). Dominance cues in nonverbal behavior. Soc. Psychol. Q..

[45] Atkinson AP, Dittrich WH, Gemmell AJ, Young AW (2004). Emotion perception from dynamic and static body expressions in point-light and full-light displays. Perception.

[46] Coulson M (2004). Attributing emotion to static body postures: Recognition accuracy, confusions, and viewpoint dependence. J. Nonverbal Behav..

[47] Hepach, R., & Tomasello M. Young children show positive emotions when seeing someone get the help they deserve. *Cogn. Dev.,* in press.

[48] Hepach R, Vaishm A, Tomasello M (2015). Novel paradigms to measure variability of behavior in early childhood: posture, gaze, and pupil dilation. Front. Psychol. Dev. Psychol..

[49] Hepach R, Vaish A, Tomasello M (2017). The fulfillment of others’ needs elevates children’s body posture. Dev. Psychol..

[50] Frewen PA, Dozois DJA, Neufeld RWJ, Densmore M, Stevens TK, Lanius RA (2011). Neuroimaging social emotional processing in women: fMRI study of script-driven imagery. Soc. Cogn. Affect. Neurosci..

[51] Tsai H-Y, Peper E, Lin I-M (2016). EEG patterns under positive/negative body postures and emotion recall tasks. NeuroRegulation.

[52] Watson D, Clark LA, Tellegan A (1988). Development and validation of brief measures of positive and negative affect: The PANAS scales. J. Pers. Soc. Psychol..

[53] Green P, MacLeod CJ (2016). simr: An R package for power analysis of generalised linear mixed models by simulation. Methods Ecol. Evol..

[54] Cumming G (2014). The new statistics: Why and how. Psychol. Sci..

[55] Parkinson B (1996). Emotions are social. Br. J. Psychol..

[56] Fessler D (2004). Shame in two cultures: Implications for evolutionary approaches. J. Cogn. Cult..

[57] Fessler, D., & Haley, K. J. The strategy of affect: Emotions in human cooperation. in *The genetic and cultural evolution of cooperation*, pp. 7–36, (2003).

[58] Frank, R. H. Cooperation through emotional commitment. in *Russell Sage Foundation series on trust: Evolution and the capacity for commitment*, Russell Sage Foundation, pp. 57–76, (2001).

[59] Keltner D, Haidt J (1999). Social functions of emotions at four levels of analysis. Cogn. Emot..

[60] Shi Y, Chung JM, Cheng JT, Tracy JL, Robins RW, Chen X, Zheng Y (2015). Cross-cultural evidence for the two-facet structure of pride. J. Res. Pers..

[61] Tracy JL, Robins RW (2007). "The nature of pride," in The self-conscious emotions: Theory and reserach.

[62] Eid M, Diener E (2001). Norms for experiencing emotions in different cultures: Inter- and intranational differences. J. Pers. Soc. Psychol..

[63] Rozin P, Royzman EB (2001). Negativity bias, negativity dominance, and contagion. Pers. Soc. Psychol. Rev..

[64] Vaish A, Grossmann T, Woodward A (2008). Not all emotions are created equal: The negativity bias in social-emotional development. Psychol. Bull..

[65] Schwartz, S. H., Sortheix, F. M. Values and subjective well-being. in *Handbook of well-being*, (Salt Lake City, UT: DEF Publishers, 2018).

[66] Oyserman D, Kemmelmeier M, Coon HM (2002). Cultural psychology, a new look: Reply to Bond (2002), Fiske (2002), Kitayama (2002), and Miller (2002). Psychol. Bull..

[67] Triandis HC, Gelfand MJ (1998). Converging measurement of horizontal and vertical individualism and collectivism. J. Pers. Soc. Psychol..

[68] Lalwani AK, Shavitt S, Johnson T (2006). What is the relation between cultural orientation and socially desirable responding?. J. Pers. Soc. Psychol..

[69] Bates D, Maechler M, Bolker B, Walker S (2015). Fitting linear mixed-effects models using lme4. J. Stat. Softw..

