# Supplementary material for: Cultural values shape the expression of self-evaluative social emotions
Source: Sci Rep. 2021 Jun 23;11:13169. doi: 10.1038/s41598-021-92652-8 (PMC8222260; doi:10.1038/s41598-021-92652-8)
Supplement: Supplementary file 1 — Supplementary Information. [file 41598_2021_92652_MOESM1_ESM.docx]

**Supplementary Information for**

**Cultural values shape the expression of self-evaluative social emotions**

Antje von Suchodoletz and Robert Hepach

Address correspondence to:

Email: avs5@nyu.edu

This PDF file includes:

1. Pre-registration details Study 1: Analysis plan

2. Pre-registration details Study 2: Analysis plan

3. Exploratory analyses

**1. Pre-registration details Study 1: Analysis plan**

**Specify exactly which analyses you will conduct to examine the main question/hypothesis.**

We will calculate liner mixed models (Gaussian error distribution) in R using the function lmer (package lme4) on the change in participants' upper-body chest height. The main predictor variables are emotion (shame, pride, joy, disappointment), cultural dimension (scaled variable extracted from the individualism/collectivism questionnaire), gender, and time after emotion induction. We expect a two-way interaction of emotion and time whilst allowing for all other possible two-way interactions of the predictor variables. In addition, we explore the combined and separate effects of gender and cultural dimension on the interaction of time and emotion. To this end we calculate two additional models with a four-way interaction of the predictor variables and one model including all three-way interactions. In each model, we include trial as a control variable, subject as a random intercept, random slopes of time on subject, random slope of trial on subject, emotion on subject, as well as random slopes for the within-subject factors interactions on subject. The last term depends on the interactions tested in the model. The statistical significance of each interaction term will be tested by comparing the full model to a reduced model without the interaction term using the drop1 function in R.

**Any secondary analyses?**

We are collecting additional data asking participants to write down the specific episode they were thinking about during the emotion induction. We plan to use these data as validation checks to our method. In addition, we are collecting data on participants' age, ethnic background, and the frequency with which they experience specific emotions. We will explore the effects of these variables on participants' postural elevation. Finally, to investigate whether the effects in our main analyses are specific to participants' upper-body posture elevation we plan to carry out the sample model comparison using the change in participants' hip height as the dependent variable.

**2. Pre-registration details Study 2: Analysis plan**

**Analyses**

(1) Pride and joy will be expressed in an elevated upper-body posture and pride will result in greater postural elevation than joy. (Statistical analysis 1a) Shame and disappointment will be expressed in a lowered upper-body posture and shame will result in lower posture than joy. (Statistical analysis 1b)

(2) We plan to combine data from Study 1 and the current Study to investigate the effects of cultural identity on the expression of both social emotions (Statistical analysis 2a) and basic emotions (Statistical analysis 2b). The cultural identity for each participant will be operationalized using confirmatory factor analysis (CFA). The CFA is run to test the factor structure in the data. Items with non-significant factor loadings will be excluded. Modification indices will be just to inform model modifications to improve model fit. Factor scores for each factor will be exported from the final model that shows good fit with the data.

(3) In a separate exploratory analysis we plan to combine data from both Study 1 and the current study to investigate whether the expression of each emotion is related to who often participants report experiencing positive and negative emotions (using the scales positive emotions and negative emotions from the PANAS (Statistical analysis 3).

(4) Based on the data of the current study we plan to investigate the effect of emotion regulation strategies on the expression of emotions (using the scales reappraisal and suppression from the Emotion Regulation Questionnaire (Statistical analysis 4) In all analyses we control for participants’ gender and age. We run each analysis for both participants' lower- and upper-body posture to follow up from initial findings that emotional expressiveness was greatest for upper-body than lower-body posture.

**Outliers and Exclusions**

We will exclude observations by trial if no data could be recorded (because of technical errors). The processing of the data proceeds along a series of predefined routines which have been developed for prior projects and which are available in OSF: https://osf.io/m4vk7/?view_only=10dbaa5378e64fee9b1d74e0246cdadb

**3. Exploratory analyses**

We analyzed whether the absolute difference in postural expression between the positive (pride) and negative (shame) social emotion can be explained by the general expected difference between positive and negative emotions. In doing so, we fitted an additional model which included the interaction of valence (positive vs. negative) and emotion category (social vs. other). The remaining model structure remained the same except that we removed the cultural variables to reduce model complexity. The resulting interaction, $\boldsymbol{\chi}^{\boldsymbol{2}}$(*df* = 1) = 9.95, *p* = .002, indicated that the difference between pride and shame could not be explained by a mere difference in valence for the emotions (i.e., positive versus negative).

We further explored whether changes in participants’ upper-body posture correlated with their PANAS-ratings of the extent to which they have felt positive and negative feelings and emotions in everyday life. These analyses were conducted with data combined from both studies. The relation between postural changes in response to the pride-prompt with the frequency of positive affect was near zero, ρ(n = 121) = .03 with a negligible effect size, η^2 = .0009. The same was true for a near-zero correlation of recalling and episode of pride in our study and experiencing negative affect in everyday life, ρ(n =121) = -.04, η^2= .0016. Furthermore, there was a positive relation between adults’ changes in posture in response to recalling joy with self-reported positive affect, ρ(n = 121) = .17, but again the effect was near zero, η^2 = .03. For the emotion of joy, the relation with the frequency of recalling negative affect was ρ(n = 121) = -.13, η^2 = .017. For recalling a disappointing emotional episode, we found, surprisingly, positive relations between postural changes and the PANAS-rating of negative affect (ρ(n = 121) = .06, η^2 = .004) as well as between postural changes and PANAS-ratings of positive affect (ρ(n = 121) = .12, η^2 = .01). A similarly counterintuitive pattern emerged for recalling an episode of shame where an increase in participants’ body posture was positively related to the frequency with which the felt negative affect (ρ(n = 121) = .09, η^2 = .008) and positive affect (ρ(n = 121) = .06, η^2 = .004) in everyday life. Overall, however, it should be noted that these were exploratory analyses which revealed near-zero effect size estimates.
